# Home Visits by Mother–Child Nurses to Concerned Mothers and Infants in Japan: Characteristics of Mothers and Infants Who Receive Continued Support

**DOI:** 10.24546/0100504448

**Published:** 2026-06-02

**Authors:** NAOKO KAWASHITA, MISA SHIOMI, RIE IKEDA, SHINOBU NOMACHI, YUKA OKAZAKI, AKIO YAMAMOTO, HISAKO IZUMI

**Affiliations:** 1 Niimi University Faculty of Human Health Sciences, Okayama, Japan; 2 Kobe University Graduate School of Health Sciences, Kobe, Hyogo, Japan; 3 Human Health Sciences, Kyoto University Graduate School of Medicine, Kyoto, Japan; 4 Wakayama Medical University School of Health and Nursing Science, Wakayama, Japan; 5 College of Nursing Art and Science, University of Hyogo, Hyogo, Japan; 6 Okayama Prefectural University Faculty of Health and Welfare Science, Okayama, Japan; 7 Kobe University Graduate School of Health Sciences (Currently Kobe University Graduate School of Medicine), Kobe, Hyogo, Japan

**Keywords:** Postpartum, Depression, Focus groups, Mother-child nurses in the community

## Abstract

**Background:**

In Japan, postnatal care is delivered through follow-up home visits conducted by mother–child nurses in the community employed by local governments. During these visits, the nurses are responsible for promptly assessing the family’s circumstances and mother’s mental health and determining appropriate directions for ongoing support. This study aimed to determine the characteristics of mothers and children assessed by mother–child nurses in the community as needing ongoing support.

**Methods:**

In this qualitative study, participants were enrolled through convenience sampling. In total, 17 mother–child nurses in the community working at three Japanese local government health centres were surveyed through focus group interviews.

**Results:**

The characteristics of mothers and children requiring ongoing support generated 11 categories and 49 subcategories, which were organised into the following six classifications: physical factors of the mother, psychological and emotional factors, socioeconomic factors, social support factors, behavioural factors and physical factors of the child.

**Discussion:**

This study revealed that mother–child nurses in the community conducted comprehensive assessments of mothers and children. Understanding their multifaceted characteristics aids in early abuse detection, postpartum depression prevention and maternal mental health promotion. The findings reveal the importance of providing seamless support for mothers and infants, offering valuable insights for mother–child nurses in the community to consider in their care practices.

## INTRODUCTION

The 2023 World Population White Paper published by the United Nations Population Fund (1) indicates that the global average total fertility rate was 2.3%, whereas that of Japan was 1.3%, which are relatively low values on a global scale. In this context, child abuse is a pervasive worldwide phenomenon (2). Child abuse poses a threat to the healthy growth and development of children. Even in Japan, where the birth rate is declining, child abuse constitutes a significant public health concern.

Local postnatal support in other countries is provided by the USA Nurse–Family Partnership (NFP) (3) and the UK Family Nurse Partnership (FNP) (4), a programme based on the NFP. The latter provides support from early pregnancy until the child is 2 years old. Both programmes are offered from the early stages of pregnancy until the child is 2 years old and up to a maximum age of 19 years or younger in some regions. The programme entails the regular visits of specially trained nurses to first-time mothers (aged 20–24 years) who are eligible for the programme. The assistance is designed to provide support to mothers with emerging problems and to identify mothers at high risk of abuse. However, these programmes may not identify mothers and infants with latent problems.

Evidently, the implementation of an outreach programme targeting potential mothers and infants in Japan is needed. In Japan, postnatal support is provided by mother–child nurses in the community (MCNCs), who are employed by local governments to make follow-up home visits after birth. The following are some of the most common assessment tools of postnatal support in Japan by Suzumiya et al. (5): (i) a parenting support checklist; (ii) the Japanese version of the Edinburgh Postnatal Depression Scale (EPDS), which was developed by Cox et al. (6) and consists of 10 items (30 points in total); and (iii) a Japanese version of Mother-to-Infant Bonding Scale (MIBS) by Yoshida et al. (7). Okano et al. (8) and Yamashita et al. (9) tested the reliability and validity of the EPDS Japanese version and standardised the cut-off point at 8/9 points (sensitivity: 0.75–0.82; specificity: 0.93–0.95). Mothers with an EPDS score of 9 or above on the three self-administered questionnaires are deemed to be at high risk of postpartum depression and are therefore offered continued support. Furthermore, the 2020 manual by the Japanese Society of Psychiatry and Neurology and the Japan Society of Obstetrics and Gynaecology (10) recommends using an EPDS cut-off score of 8/9 and emphasises on the importance of closely monitoring cases where item 10 scores ≥1. Matsunaga et al. (11) identified the MIBS as a valuable tool for screening attachment disorders, with an optimal cut-off of 4/5 points (sensitivity: 0.85–0.90; specificity: 0.79–0.90) at 1 month postpartum. Conversely, the parenting support checklist lacks a defined cut-off, making it difficult to determine clear criteria for continued support. Therefore, during home visits, Japanese MCNCs must promptly assess each family’s circumstances, maternal mental health and the appropriate course of ongoing support. The decision to continue is then left to the discretion of the MCNC. However, judgements may vary according to the MCNCs skills and experience. Tamura et al. (12) posited that public health nurses must possess the practical skills to ‘capture the actual living conditions and health problems of the population from the perspective of public health and disease prevention’. In contrast, Wilson et al. (13) asserted that ‘home visits’ are highly crucial in assessing the mother–infant relationship. MCNCs must maximise the opportunity in home visits to anticipate potential health issues affecting mothers and infants and to provide interventions, as needed to identify mothers and infants in need of continued support at an early stage. Consequently, the mothers and infants that MCNCs support need to be identified to provide them continued assistance through home visits.

Several studies have investigated the effectiveness of home visits (13–15). However, there is a paucity of national and international literature focusing on the assessment of MCNCs during home visits (16). In practice, decisions on continuing support during home visits are often based on MCNCs’ individual judgement, which may vary with their professional experience and skills. Although standardised screening tools such as the EPDS are widely used, they may not capture latent or multifaceted concerns observed during home visits.

Experienced MCNCs can better integrate multiple cues—such as mothers’ behaviours, emotional expressions, family interactions and living environments—into their assessments. However, these nuanced perspectives have rarely been explicitly described or systematically organised. Therefore, this study qualitatively explored and clarified the characteristics of mothers and infants who required continued support, as assessed by MCNCs, including those at risk of losing access to support due to low EPDS scores. By making these assessment perspectives explicit and categorising them within a shared framework, this study contributes to the development of more objective and consistent assessments that are less dependent on individual experience or MCNCs’ skills. Reliable assessments will facilitate the accumulation of essential information and development of preventative interventions for mothers in need of continued support, thereby ensuring the seamless provision of parenting support services in Japan.

## MATERIALS AND METHODS

### Design

This study adopted a qualitative design. Focus group interviews were conducted as the most appropriate research method. Focus group interviews are relevant and applicable to exploratory research aimed at elucidating somewhat unknown concepts rather than measuring predetermined variables (17, 18).

### Participants

The study was conducted using a convenience sampling method. The participants were selected based on the following criteria:

- Have conducted home visits with mothers and infants for at least 1 year;
- Working as full-time or part-time public health nurse or midwife; and
- Experienced in providing ongoing support through new-born follow-up visits. Individuals who did not meet the inclusion criteria were excluded from the study.

A minimum of 1 year of experience in conducting home visits was established. This ensured that participants had sufficient exposure to a full annual cycle of maternal and child health services and had developed stable assessment practices based on accumulated experience, rather than immediate deployment during the initial period following appointment or transfer. This criterion is consistent with qualitative research methodology, which emphasises the importance of participants’ experiential knowledge when exploring professional judgement (17, 18).

The managers of three local authority health centres were contacted and informed of the objectives and methodology of the study. They were then asked to refer for the study five to seven MCNCs (17 in total) who met the inclusion criteria. Note that research participants are assigned from A to Q.

### Procedure and data collection

The study period commenced on 1 January, 2020 and concluded on 31 March, 2021. All participants were interviewed at their workplace. Each of the three focus group interviews lasted 60–90 minutes and were conducted by two researchers, with the primary interviewer consistently conducting the interviews and the deputy interviewer posing supplementary questions where necessary to ensure uniformity and transparency. The interviews were conducted either in person or remotely via video conferencing. The interviews were either recorded with an integrated circuit recorder or, in the case of online interviews, recorded and transcribed, with the consent of the participant. Each group participated in a single interview session.

Six female researchers (HI, MS, RI, NS, YO and NK) conducted the interviews. The interviewers held doctoral degrees in various disciplines: HI (Doctor of Medicine); MS and RI (Doctor of Health Sciences); NS (Doctor of Nursing Science); YO (Doctor of School Education) and NK, a maternal nursing researcher from Niimi University. HI and MS brought substantial experience in facilitating focus group interviews, whereas the remaining researchers, though less experienced, underwent thorough training and received detailed guidance before conducting sessions. None of the researchers had pre-existing relationships with the participants, and no characteristics were identified that may have influenced participant responses. No conflicts of interest were reported.

Semi-structured interview guide was developed to facilitate the focus group interviews. The interview guide consisted of a series of conversational statements, including an introduction, a description and practicalities of the group interview, precautions and key and concluding questions. The interviews were conducted in accordance with the following protocol: (Appendix)

- Please recall a mother and infant who had an EPDS score <9 at the time of the visit but continued to receive support. What specific aspects were of concern?
  ① What were the mother’s behavioural, linguistic and facial expressions?
  ② What are your thoughts on the child’s condition?
  ③ How are the infant’s older siblings doing?
  ④ What concerns do you have about the inside and outside of the house?
- What factors are most important to you in determining whether to continue providing support during the visit?

Notably, at the commencement of the interview, a questionnaire was administered to ascertain the MCNC’s age, sex, qualifications obtained, years of experience in nursing, years of experience as a MCNC and the number of visits to the new-born in the previous year.

### Ethical considerations

The purpose of the study was clearly articulated in writing and verbally to the management of the health centres. After consent to participate in the study was obtained from the manager, subjects were referred to the study by the manager. Those who consented to participate in the study were provided with written information about the purpose of the study, their right to refuse and the protection of their privacy. This study was approved by the research ethics committee of the Okayama Prefectural University, with which the first author has been affiliated since 22 November, 2019 (Number: 19–73).

### Data analysis

Qualitative data were analysed inductively through a three-stage process to ensure the trustworthiness of the findings, following established qualitative research methodology (17, 18).

In the first stage, interviewers from three teams (HI & NK, MS & SN and YO & RI) reviewed the interview transcripts and extracted sentences and phrases that reflected ‘characteristics of mothers and children that MCNCs care about’. In the second stage, the extracted content was grouped into subcategories and broader categories based on similarities and differences in meaning. All researchers engaged in repeated discussions to maintain consistency and rigour in the coding process. Categories and subcategories were continuously refined, and data saturation was considered reached when no new themes emerged. In the third stage, the finalised categories were further organised into higher-order classifications to reflect the broader conceptual domains underlying MCNCs’ assessments. These classifications were developed through iterative discussions within the research team to enhance conceptual clarity and analytical coherence. To enhance reliability, member checks were conducted with all participants. No significant changes or additional feedback were received, and participants confirmed agreement with the analysis. Finally, NK applied the finalised subcategories, categories and classifications across the entire dataset, with verification performed by HI.

## RESULTS

### Demographics

The attributes of the research participants are shown in Table I. The 17 participants in this study were women in their 20s–50s, with most of them (52.9%) in their 40s. Eleven (64.8%) participants were public health nurses, three (17.6%) were midwives and three (17.6%) were midwives and public health nurses.

### Overview of qualitative findings

Analysis of the interviews yielded 49 subcategories and 11 categories, which were then sorted into 6 classifications (Tables II-1–II-6). Detailed subcategories and representative quotations are presented in Appendices A–F. Although most subcategories were reflected in the accounts of multiple participants, some were represented by a single quotation that reflected analytically important perspectives, even though they were mentioned infrequently. Notably, these subcategories emerged through systemic coding and comparative analysis of multiple data segments and were subsequently validated through member checking.

#### Classification 1: Physical factors of the mother

One category of physical factors for mothers of concern was generated from four subcategories.

##### Category 1: Concerns about the mother’s health

This category was generated from four subcategories: ‘advanced maternal age’, ‘poor postpartum health’, ‘insufficient rest or sleep’ and ‘inadequate nutrition’.

#### Classification 2: Psychological and emotional factors

The three categories of psychological and spiritual factors of concern were generated from 19 subcategories.

##### Category 1: Possessing qualities that predispose them to unstable mental states

This category consisted of seven subcategories: ‘history or current mental illness (including suspected mental illness)’, ‘suspected intellectual or developmental disabilities’, ‘emotional instability’, ‘low self-esteem’, ‘strong childcare anxiety’, ‘nervous disposition’ and ‘overworking personality’.

##### Category 2: Not expressing their feelings

This category was generated from three subcategories: ‘unable to express their feelings’, ‘discrepancies in EPDS scores’ and ‘poor facial expressions and responses’.

##### Category 3: Negative views of pregnancy, childbirth and childcare

This category was generated from nine subcategories: ‘unplanned pregnancy’, ‘low satisfaction with childbirth’, ‘negative birth experience’, ‘aversion to breastfeeding’, ‘gap between ideal parenting beliefs and reality’, ‘difficulty with raising their infants’, ‘lack of enjoyment in childcare’, ‘difficulty caring for the infant alongside older siblings’ and ‘child-rearing stress because of the infant’s older siblings’.

Regarding parenting, MCNCs frequently identified mothers’ negative feelings stemming from a discrepancy between idealised expectations formed during pregnancy—such as ‘babies are cute’ or ‘breastfeeding will naturally be established after birth’—and the reality of postnatal child-rearing. When these expectations were not met, they were perceived as contributing to maternal distress and difficulties in child-rearing.

#### Classification 3: Socioeconomic factors

A single category under socioeconomic factors of concern was generated from seven subcategories.

##### Category 1: Social high-risk factors

This category was generated from seven subcategories: ‘high educational attainment’, ‘economic constraints’, ‘lack of family registration’, ‘young maternal age’, ‘foreign citizenship’, ‘abused’ and ‘lost relatives’.

#### Classification 4: Social support factors

The two categories of social support factors of concern were generated from seven subcategories.

##### Category 1: Concerned about support from partner or family

This category was generated from three subcategories: ‘lack of partner and family support’, ‘dissatisfied with partner or family support’ and ‘poor relationship with partner, biological parents or parents-in-law’.

##### Category 2: Not receiving or asking support from others

This category was generated from four subcategories: ‘difficulty asking for support’, ‘not accepting support’, ‘difficulty socialising’ and ‘lack of friends or someone to consult’.

#### Classification 5: Behavioural factors

The three categories of behavioural factors of concern were generated from eight subcategories.

##### Category 1: Uncomfortable with mother’s parenting behaviour

This category was generated from three subcategories: ‘clumsy parenting behaviour’, ‘inappropriate parenting behaviour’ and ‘inadequate parenting behaviour towards the infant’s older siblings’.

##### Category 2: Inappropriate motherly behaviour

This category was generated from three subcategories: ‘unable to provide a nurturing environment’, ‘uncomfortable in behaviour and dress’ and ‘prioritising pets over the infant’.

##### Category 3: Inability to maintain personal appearance

This category was generated from two subcategories: ‘mother’s inability to take care of her own personal appearance’ and ‘appearance suggesting financial strain’.

#### Classification 6: Physical factors of the child

One category of physical factors for children of concern was generated from four subcategories.

##### Category 1: Concerns about the child’s health

This category was generated from four subcategories: ‘the infant has (or is suspected of having) a disease’, ‘preterm and low-birth-weight infants’, ‘not gaining weight’ and ‘the infant’s older sibling has developmental delays’.

## DISCUSSION

This study has shown that MCNCs provide ongoing support to mothers and children of concern by assessing not only the risk of postpartum depression with an EPDS score cut-off of 9 or higher, but also information on the mother’s physical factors, behavioural factors, socioeconomic factors, social support factors, psychological and emotional factors and the child’s physical factors from multiple perspectives.

The NFP (3) and FNP (4) implemented in other countries provide continuous support to young first-time mothers aged <19 years and socioeconomically disadvantaged mothers under aged <24 years (low income, homeless, etc.) and for their new-born, from birth up to 2 years of age. Similarly, in Japan, MCNCs conduct antenatal interviews in early pregnancy in order to distribute the Maternal and Child Health Handbook (Ministry of Health, Labour and Welfare). The antenatal interview aims to provide seamless support from pregnancy to childcare, and MCNCs use the information collected during the antenatal interview and postnatal home visits to determine whether further support is needed, focusing on various social risks (unplanned pregnancy, foreign nationality, etc.) and various socioeconomic factors to determine whether or not further support was needed.

In particular, Japanese MCNCs focus primarily on social support status. In Japan, although fathers have been encouraged to participate in childcare in recent years, childcare within the family is often the responsibility of the mother (19). Webster et al. (20) and Iliadis et al. (21) found that fathers and mothers who receive less support from their families are at higher risk of postpartum depression. Less support also means that mothers are more likely to experience physical and emotional exhaustion and negative emotions since they take on many roles. Therefore, social support that satisfies mothers’ needs must be present, such as those from their husbands and family members. The MCNCs were also concerned about mothers who were unable to ask for support because of concern for their children’s fathers, mothers who did not accept social support and mothers who had difficulty socialising with others. Iwata et al. (22) found that assessing mothers’ difficulties in asking for help could help increase social support. The current state of social support and mothers’ perceptions of social support must be assessed.

In terms of psychological and emotional factors for mothers, support is provided in countries other than Japan as there is a risk of postpartum depression if above the EPDS cut-off. Netsi et al. (23) found that children of mothers with severe and persistent postpartum depression have negative developmental outcomes. In Japan, ongoing support is provided if the mother scores above the EPDS cut-off. MCNCs judged the mental health status of mothers not only based on their history and current mental illness (including suspected mental illness), but also on the presence of their psychological and emotional characteristics, their views of pregnancy, childbirth and childcare as a negative experience and whether they needed further support. MCNCs therefore need to focus on psychological and emotional factors other than the risk of postpartum depression. Tanabe (24) reported that vaginal birth is broadly classified as ‘natural birth’ and obstetric interventions, such as caesarean section and painless birth, as ‘unnatural birth’. According to Japan’s Ministry of Health, Labour and Welfare (25), approximately 70% of mothers underwent a ‘natural birth’ in 2020. Therefore, presumably, if a birth in Japan had had obstetric intervention, the experience would have been negative and the birth would have been considered unnatural. Regarding parenting, negative feelings clearly arose from the gap between ideal parenting beliefs held during pregnancy and actual parenting experience, such as ‘babies are cute’ and ‘breastfeeding can be done after the baby is born’. Multiparous mothers were also assessed for difficulties and stress in caring for the infant’s siblings since they had to care for their other children in addition to caring for their new-born. The mother’s inability to adequately care for her infant’s siblings, which she had been able to do in the past, may have contributed to her negative feelings. Kimura, N. and Hotta (26) reported that self-affirmation is increased by, for example, making a gentle effort for the sake of the child and the family, which further promotes the maternal role. Regardless of whether mothers are primiparous or multiparous, MCNCs should check that mothers do not view childbirth and childcare as a negative experience. If mothers do see childbirth and childcare negatively, MCNCs should consider recognising mothers who are doing their best in their ongoing involvement, working with them to increase their sense of self-worth and creating opportunities for peer support.

MCNCs also assess the mother’s physical factors, such as her health and whether she was getting enough rest and sleep. In Japan, 54.4% of mothers sleep with their children at home (27). Problems with mothers experiencing sleep problems due to infant night waking and their responses have also been noted (28). Thus, MCNCs must identify and help alleviate fatigue resulting from maternal postpartum health and accumulated sleep deprivation.

Herein, Japanese MCNCs were also concerned about maternal behavioural factors, such as their parenting behaviour towards their infants and siblings and the parenting environment. In recent years, the declining birth rate and the shift to nuclear families in Japan have resulted in fewer opportunities for mothers to interact with infants, and the number of mothers with no parenting experience is increasing. Such Mothers have little parenting knowledge, making it difficult for them to change their parenting practices according to their children’s development. Yokoyama et al. (29) noted that some mothers feel that parenting practices should be improved, but give up or experience difficulties and say that they do not implement them because they are too tedious. Moreover, in the present study, for mothers who did not raise their infants or other children well or did not provide a nurturing environment, the mother’s temperament, in addition to her lack of knowledge and experience, possibly hindered her ability to provide care accordingly. Takeda and Kobayashi, (30) found that the temperament of mothers were crucial to proper childcare, and MCNCs need to view the mother’s temperament as an indicator of judgement.

Japanese MCNCs were also concerned about the infant’s physical factors. Ueda et al. (31) noted that infant health problems can be a stressor for the mother. They suggest that if the infant’s condition deviates from normal expectations, such as hospitalisation, this may increase maternal anxiety and lead to the emergence of depressive symptoms. MCNCs therefore need to identify potential problems in the child and intervene early.

The results of this study have shown that MCNCs assessed mothers and infants from multiple perspectives and decided to support their mental health and their infant’s growth and development. The characteristics of mothers and infants of concern identified in this study will facilitate the assessment of mothers and infants with potential problems and those who are vulnerable to interruptions in support. These characteristics are important when considering ‘seamless support’, which is expected promote healthy infant growth and development.

Furthermore, the cases identified in this study as mothers and children of concern could not simply be categorised between postpartum depression and abuse; rather, the categorisation is multifaceted, capturing the characteristics of both mothers and children. We believe that home visits by MCNCs in Japan supports not only the early identification of abuse but also the prevention of postpartum depression and declining maternal mental health.

Apart from young first-time mothers aged <19 years and socioeconomically disadvantaged mothers aged <24 years (low income, no housing, unmarried or not living with a partner for more than 1 year, low education, etc.), who are the target of NFP, FNP and other programmes in other countries, the mothers and infants identified by the MCNCs as needing continued support in this study were assessed from a multifaceted perspective. However, this study has several limitations. First, the study only surveyed 17 MCNCs, which may not be sufficiently representative of MCNCs in Japan. Second, the MCNCs had a wide range of experience, from 1 to 19 years, which may have influenced the participants’ responses. Further investigation involving larger datasets is necessary to develop an assessment tool for MCNCs to objectively assess the need of new mothers and infants for continued support in their local communities.

## Figures and Tables

**Table I:** Characteristics of mother–child nurses in the community (n = 17)

		n (%)	Mean [range]
Age (years)	20s	2 (11.8)	
	30s	2 (11.8)	
	40s	9 (52.9)	
	50s	4 (23.5)	

Qualifications acquired	Public health nurse	11 (64.8)	
	Midwife	3 (17.6)	
	Midwife and public health nurse	3 (17.6)	

MCNC experience (years)			6 [1–19]
Nursing experience (years)			16 [3–31]
Total number of visits last year (cases)			30 [4–150]

MCNC, mother–child nurses in the community.

**Table II-1:** Characteristics of the mothers and children of concern identified by MCNCs (physical factors of the mother)

Classification	Category	Subcategory	Representative data (Study participant IDs)
Physical factors of the mother	Concerns about the mother’s health	Advanced maternal age	C, O
		Poor postpartum health	A, N
		Insufficient rest or sleep	P, Q
		Inadequate nutrition	M

MCNCs, mother–child nurses in the community.

**Table II-2:** Characteristics of the mothers and children of concern identified by MCNCs (psychological and emotional factors)

Classification	Category	Subcategory	Representative data (Study participant IDs)
Psychological and emotional factors	Possessing qualities that predispose them to unstable mental states	Previous or current mental illness (including suspected mental illness)	A, D, M
		Suspected intellectual or developmental disabilities	B, C, D, F, G, I, J
		Emotional instability	B, C, D, F, L, M, N, P
		Low self-esteem	P, Q
		Strong child care anxiety	A, B, C, D, G, I, J, L, M, N, O
		Nervous disposition	B, M, N, P, Q
		Overworking personality	I, O, P
	Not expressing their feelings	Unable to express their feelings	G, H, I. K, P
		Discrepancies in EPDS scores	I, J, K, L
		Poor facial expressions and responses	C, D, L, M, O, P, Q
	Negative views of pregnancy, childbirth and childcare	Unplanned pregnancy	D, E, I
		Low satisfaction with childbirth	O, P
		Negative birth experience	O, P, Q
		Aversion to breastfeeding	C, D
		Gap between ideal parenting beliefs and reality	I, O
		Difficulty with raising their infants	J, M, O
		Lack of enjoyment in childcare	F, M
		Difficulty caring for the infant alongside older siblings	M, O
		Child-rearing stress because of the infant’s older siblings	A, F

MCNCs, mother–child nurses in the community; EPDS, Edinburgh Postnatal Depression Scale.

**Table II-3:** Characteristics of the mothers and children of concern identified by MCNCs (socioeconomic factors)

Classification	Category	Subcategory	Representative data (Study participant IDs)
Socioeconomic factors	Social high-risk factors	High educational attainment	I, O
		Economic constraints	B, C, D, N
		Lack of family registration	D, E
		Young maternal age	C, D, G
		Foreign citizenship	D, F, H
		Abused	D, M
		Lost relatives	B

MCNCs, mother–child nurses in the community.

**Table II-4:** Characteristics of the mothers and children of concern identified by MCNCs (social support factors)

Classification	Category	Subcategory	Representative data (Study participant IDs)
Social support factors	Concerns about support from partner or family	Lack of partner or family support	A, B, D, E, K, M, N
		Dissatisfied with partner or family support	M, N, O, P
		Poor relationship with partner, biological parents or parents-in-law	G, M, Q
	Not receiving or asking support from others	Difficulty asking for support	O, P, Q
		Not accepting support	A, B, C
		Difficulty socialising	A, C, D, E, F, J, K, L, O, Q
		Lack of friends or someone to consult	E, K, P

MCNCs, mother–child nurses in the community.

**Table II-5:** Characteristics of the mothers and children of concern identified by MCNCs (behavioural factors)

Classification	Category	Subcategory	Representative data (Study participant IDs)
Behavioural factors	Uncomfortable with mother’s parenting behaviour	Clumsy parenting behaviour	A, B, D, F, H, M, N, O, P, Q
		Inappropriate parenting behaviour	A, B, C, D, F, H, K
		Inadequate parenting behaviour towards the infant’s older siblings	F, J
	Inappropriate motherly behaviour	Unable to provide a nurturing environment	A, C, D, E, F, H, I, J, K, L, M, Q
		Uncomfortable in behaviour and dress	A, B, C, D, F, J, L
		Prioritising pets over the infant	C, F
	Inability to maintain personal appearance	Mother’s inability to take care of her own personal appearance	H, I, M
		Appearance suggesting financial strain	I, M

MCNCs, mother–child nurses in the community.

**Table II-6:** Characteristics of the mothers and children of concern identified by MCNCs (physical factors of the child)

Classification	Category	Subcategory	Representative data (Study participant IDs)
Physical factors of the child	Concerns about the child’s health	The infant has (or is suspected of having) a disease	F, I, J, K, L
		Preterm and low-birth-weight infants	J, M
		Not gaining weight	C, D, F, G, L, M, O
		Concern about developmental delays in the infant due to delays in an older sibling	C, M

MCNCs, mother–child nurses in the community.

## Appendices

**Table:**

**A**. Physical factors of the mother		
Category	Subcategory	Participant Voice
Concerns about the mother’s health	Advanced maternal age	Being an older mother was something that stood out during the visit. (Participant C)
	Poor postpartum health	One mother still had an open perineal wound after childbirth, and she seemed physically quite vulnerable. (Participant A)
	Insufficient rest or sleep	She looked very tired because she was waking up many times at night and was not getting enough rest. (Participant Q)
	Inadequate nutrition	One mother said she was not really eating proper meals because she was too busy. (Participant M)

**Table:**

**B**. Psychological and emotional factors		
Category	Subcategory	Participant Voice
Possessing qualities that predispose them to unstable mental states	Previous or current mental illness (including suspected mental illness)	Mothers who had experienced mental difficulties before pregnancy or had a history of mental illness were mentioned. Some had seemed fine during pregnancy, but after childbirth, things appeared to have become very difficult for them. (Participant M)
	Suspected intellectual or developmental disabilities	Some mothers did not have a formal diagnosis, but I felt they might have developmental disabilities or otherwise found communication difficult. (Participant D)
	Emotional instability	One mother said that when she looked at social networking sites, she wondered why everyone else seemed to be managing well while she felt exhausted. (Participant N)
		During one visit, I witnessed clear mood swings within a short time. (Participant L)
	Low self-esteem	One mother said she had very low self-esteem and always put her husband and family before herself. (Participant Q)
	Strong childcare anxiety	Some mothers repeatedly searched online for childcare information and appeared unable to manage their anxiety. (Participant M)
		Even after I explained things, she still seemed anxious and continued asking questions. (Participant D)
	Nervous disposition	One mother repeatedly told me exactly how much breast milk she had expressed and how much the baby had taken. She kept checking the baby’s weight for months. (Participant B)
		Another mother seemed very worried about disturbing the neighbours if her baby cried. (Participant Q)
	Overworking personality	Some mothers said they felt they had to do everything by themselves and ended up sacrificing sleep. (Participant P)
		One mother said she had a clear idea of how parenting should be done and felt like she was failing when things did not go that way. (Participant O)
Not expressing their feelings	Unable to express their feelings	Some mothers said they were fine and were not having any problems, but actually they seemed unable to talk about what was really troubling them. (Participant H)
	Discrepancies in EPDS scores	Some mothers had low EPDS scores, and I wondered whether they were marking them lower than how they really felt. (Participant I)
		Mothers who scored zero also worried me because I felt they might not have been expressing their anxiety appropriately. (Participant K)
	Poor facial expressions and responses	One mother rarely smiled and showed very little reaction during our visit. (Participant D)
		One mother watched from a distance while I interacted with her baby. She looked like as if she was watching as a detached third person. (Participant C)
Negative views of pregnancy, childbirth and childcare	Unplanned pregnancy	Some mothers said the pregnancy had been unwanted, and I felt we needed to follow them more closely. (Participant I)
		In some cases, the pregnancy had not been reported. (Participant D)
	Low satisfaction with childbirth	Some mothers talked about feeling disappointed with their childbirth experience, such as not being able to share the moment with their partner. (Participant P)
	Negative birth experience	Some mothers expressed guilt about obstetric procedures or premature birth, even though they had been told it was not their fault. (Participant Q)
	Aversion to breastfeeding	One mother said she felt uncomfortable holding or breastfeeding her baby. (Participant C)
	Gap between ideal parenting beliefs and reality	Some mothers said they thought everything would go smoothly, and they were confused when it did not. (Participant O)
	Difficulty with raising their infants	Some babies slept very little, cried often or resisted being held, and their mothers looked exhausted (Participant J)
	Lack of enjoyment in childcare	When mothers said childcare was ‘rarely’ or ‘almost never’ enjoyable, I felt concerned. (Participant F)
	Difficulty caring for the infant alongside older siblings	Some mothers said they did not know what to do when both their infant and older child needed them at the same time. (Participant M)
	Child-rearing stress because of the infant’s older siblings	The mother appeared affectionate toward the baby but sometimes seemed irritated with the older sister. (Participant A)
		I sometimes saw mothers speak harshly to older children when they were under a lot of stress. (Participant F)

**Table:**

**C**. Socioeconomic factors		
Category	Subcategory	Participant Voice
Social high-risk factors	High educational attainment	Some highly educated mothers could not easily admit they were having difficulties. (Participant I)
	Economic constraints	Some mothers said they had housing loans and felt the added burden. (Participant N)
	Lack of family registration	Some mothers were having trouble with registration. (Participant D)
	Young maternal age	Some young mothers became unreachable after pregnancy, even though they had responded during earlier interviews. (Participant C)
	Foreign citizenship	Differences in childcare practices related to cultural background sometimes created further communication difficulties. (Participant F)
	Abused	Some mothers had experienced abuse themselves, and I sometimes saw them speaking harshly to their children. (Participant M)
	Lost relatives	Some mothers were still grieving the loss of a close relative, and this seemed to visibly affect their postpartum state. (Participant B)

**Table:**

**D**. Social support factors		
Category	Subcategory	Participant Voice
Concerns about support from partner or family	Lack of partner or family support	Some mothers were caring for their babies mostly on their own and were not getting enough rest. (Participant M)
		Some said their partners did not really understand how distressed they felt. (Participant N)
	Dissatisfied with partner or family support	When family members gave advice, some mothers seemed to feel more anxious. (Participant O)
	Poor relationship with partner, biological parents or parents-in-law	Some partners and parents would say, ‘Tell me and I’ll do it’, and did not act unless they were told what to do. This left the mothers exhausted and frustrated. (Participant Q)
		One mother said that her relationship with her birth mother was not going well. She had told her that she had given birth but did not allow her to meet the baby. (Participant M)
Not receiving or asking support from others	Difficulty asking for support	Some mothers said that when their partners came home tired from work, they felt bad about talking about their own concerns. (Participant P)
	Not accepting support	One participant said that although family members—such as the mother’s own mother, father and grandmother—or members of the community who could support her were present, she did not accept their support. (Participant C)
	Difficulty socialising	I thought that those who could build connections on their own would probably be fine. I was concerned about those who could not actively do so. (Participant O)
	Lack of friends or someone to consult	I was concerned about mothers who seemed isolated or who said they had no friends around them. (Participant K)
		When I visited one mother, she said it had been a long time since she had spoken with another adult, and she talked nonstop as if a dam had burst. She said there was no one around who would fully listen to her. (Participant P)

**Table:**

**E**. Behavioural factors		
Category	Subcategory	Participant Voice
Uncomfortable with mother’s Parenting behaviour	Clumsy parenting behaviour	I was concerned about mothers who were unable to adjust their child care strategy as their infant grew or adapt to the situation. (Participant P)
	Inappropriate parenting behaviour	I saw cases where mothers did not change their child’s clothes after vomit or other stains or did not replace towels that were wet with urine. (Participant K)
	Inadequate parenting behaviour towards the infant’s older siblings	I noticed that some siblings were wearing clothes that were out of season or dirty or were dressed only in their underwear. (Participant F)
Inappropriate motherly behaviour	Unable to provide a nurturing environment	One mother was nicely made up, but her house was not tidy. Things were left on the table. (Participant H)
	Uncomfortable in behaviour and dress	The mother appeared very clean, but the child had dirt on her. I observed a difference between the appearance of the mother and that of the infant. I was concerned that her daily life might not be centred on the infant. (Participant F)
		I was worried about how she would care for her infant with such decorative nails. (Participant L)
	Prioritising pets over the infant	They did not seem focused on the infant and appeared worried about their pets. They worried more about how to care for their pets postpartum. (Participant C)
Inability to maintain personal appearance	Mother’s inability to take care of her own personal appearance	I was concerned about mothers who came out wearing clothes that looked like pyjamas when visitors arrived. (Participant I)
	Appearance suggesting financial strain	She seemed busy every day and looked like she did not have much room to relax. Her facial expression looked tired. She was not rejecting her child, but she seemed busy and was sometimes difficult to reach even when we contacted her. (Participant M)

**Table:**

**F**. Physical factors of the child		
Category	Subcategory	Participant Voice
Concerns about the child’s health	The infant has (or is suspected of having) a disease	The infant did not move their arms and legs much. They appeared vacant or did not cry. Mothers were often told that the baby was ‘easy’ because they did not cry, but the baby’s whole body felt soft and listless. (Participant L)
	Preterm and low-birth-weight infants	Some infants were born prematurely and had been in the hospital for 2 months. The mother also appeared to be carrying a heavy burden. (Participant M)
	Not gaining weight	The infant did not drink breast milk or formula well. Because of this, weight gain was not evident, and the mother was worried. She was trying her best to breastfeed, but the baby did not drink much. In those cases, I was concerned. (Participant O)
	The infant’s older sibling has developmental delays	It might have been due to inexperience, but the infant’s older siblings seemed to speak slowly. I was concerned that this baby might also have developmental delays. (Participant M)
